# A protective maternal nutrient concomitant intake associated with acute leukemia might be modified by sex, in children under 2 years

**DOI:** 10.3389/fonc.2023.1239147

**Published:** 2023-09-07

**Authors:** Ángel Mérida-Ortega, María Luisa Pérez-Saldivar, Laura E. Espinoza-Hernández, Elisa M. Dorantes-Acosta, José Refugio Torres-Nava, Karina A. Solís-Labastida, Rogelio Paredes-Aguilera, Martha M. Velázquez-Aviña, Rosa Martha Espinosa-Elizondo, M. Raquel Miranda-Madrazo, Ana Itamar González-Ávila, Luis Rodolfo Rodríguez-Villalobos, Juan José Dosta-Herrera, Javier A. Mondragón-García, Alejandro Castañeda-Echevarría, M. Guadalupe López-Caballero, Sofía I. Martínez-Silva, Juan Rivera-González, Norma Angélica Hernández-Pineda, Jesús Flores-Botello, Jessica Arleet Pérez-Gómez, María Adriana Rodríguez-Vázquez, Delfino Torres-Valle, Jaime Ángel Olvera-Durán, Annel Martínez-Ríos, Luis R. García‐Cortés, Carolina Almeida-Hernández, Janet Flores-Lujano, Juan Carlos Núñez-Enríquez, Minerva Mata-Rocha, Haydeé Rosas-Vargas, David Aldebarán Duarte-Rodríguez, Silvia Jiménez-Morales, Juan Manuel Mejía-Arangure, Lizbeth López-Carrillo

**Affiliations:** ^1^ Center of Population Health Research, Instituto Nacional de Salud Pública (INSP), Cuernavaca, Mexico; ^2^ Unidad de Investigación Médica en Epidemiología Clínica, Hospital de Pediatría, Centro Médico Nacional (CMN) Siglo-XXI, Instituto Mexicano del Seguro Social (IMSS), México City, Mexico; ^3^ Servicio de Hematología Pediátrica, Hospital General “Gaudencio González Garza”, CMN “La Raza”, IMSS, Mexico City, Mexico; ^4^ Departamento de Hemato-Oncología, Hospital Infantil de México Federico Gómez, Secretaria de Salud (SSA), Mexico City, Mexico; ^5^ Servicio de Oncología, Hospital Pediátrico Moctezuma, Secretaría de Salud de la Ciudad de México (SSCDMX), Mexico City, Mexico; ^6^ Servicio de Hematología, UMAE Hospital de Pediatría, CMN “Siglo XXI”, IMSS, Mexico City, Mexico; ^7^ Servicio de Hematología, Instituto Nacional de Pediatría (INP), SSA, Mexico City, Mexico; ^8^ Servicio de Onco-Pediatría, Hospital Juárez de México, SSA, Mexico City, Mexico; ^9^ Servicio de Hematología Pediátrica, Hospital General de México, SSA, Mexico City, Mexico; ^10^ Servicio de Hematología Pediátrica, CMN”20 de Noviembre”, Instituto de Seguridad Social al Servicio de los Trabajadores del Estado (ISSSTE), Mexico City, Mexico; ^11^ Servicio de Hematología Pediátrica, HGR No. 1 “Dr. Carlos Mac Gregor Sánchez Navarro” IMSS, Mexico City, Mexico; ^12^ Servicio de Pediatría, Hospital Pediátrico de Tacubaya, SSCDMX, Mexico City, Mexico; ^13^ Servicio de Cirugía Pediátrica, Hospital General “Gaudencio González Garza”, CMN “La Raza”, IMSS, Mexico City, Mexico; ^14^ Servicio de Cirugía Pediátrica, Hospital General Regional (HGR) No. 1 “Dr. Carlos Mac Gregor Sánchez Navarro” IMSS, Mexico City, Mexico; ^15^ Servicio de Pediatría, Hospital General de Zona Regional (HGR) No. 25 IMSS, Mexico City, Mexico; ^16^ Coordinación Clínica y Pediatría, Hospital Pediátrico de Coyoacán, SSCDMX, Mexico City, Mexico; ^17^ Hospital Pediátrico de Iztapalapa, SSCDMX, Mexico city, Mexico; ^18^ Hospital General Dr. “Gustavo Baz Prada”, Instituto de Salud del Estado de México (ISEM), State of Mexico, Mexico; ^19^ Coordinación Clínica y Pediatría del Hospital General de Zona 76 IMSS, Ecatepec de Morelos, State of Mexico, Mexico; ^20^ Coordinación Clínica y Pediatría, Hospital General “La Perla” ISEM, Nezahualcóyotl, State of Mexico, Mexico; ^21^ Coordinación Clínica y Pediatría, HGR No. 72 “Dr. Vicente Santos Guajardo”, IMSS, Tlalnepantla de Baz, State of Mexico, Mexico; ^22^ Coordinación Clínica y Pediatría del Hospital General de Zona 68, IMSS, Ecatepec de Morelos, State of Mexico, Mexico; ^23^ Coordinación Clínica y Pediatría del Hospital General de Zona 71, IMSS, Chalco de Díaz Covarrubias, State of Mexico, Mexico; ^24^ Servicio de Cirugía Pediátrica, HGR 1° Octubre, ISSSTE, Mexico City, Mexico; ^25^ Hospital Regional “General Ignacio Zaragoza”, ISSSTE, Mexico City, Mexico; ^26^ Delegación Regional Estado de México Oriente, IMSS, Naucalpan de Juárez, State of Mexico, Mexico; ^27^ Hospital General de Ecatepec “Las Américas”, ISEM, Ecatepec de Morelos, State of Mexico, Mexico; ^28^ Laboratorio de Biología Molecular de las Leucemias, Unidad de Investigación en Genética Humana, UMAE, Hospital de Pediatría, CMN “Siglo XXI”, IMSS, Mexico City, Mexico; ^29^ Laboratorio de Genética, Hospital de Pediatría, Centro Médico Nacional (CMN) Siglo-XXI, Instituto Mexicano del Seguro Social (IMSS), Mexico City, Mexico; ^30^ Laboratorio de Innovación y Medicina de Precisión, Núcleo A, Instituto Nacional de Medicina Genómica (INMEGEN), México City, Mexico; ^31^ Laboratorio de Genómica del Cáncer, Instituto Nacional de Medicina Genómica (INMEGEN), México City, Mexico; ^32^ Facultad de Medicina, Universidad Nacional Autónoma de México (UNAM), México City, Mexico

**Keywords:** leukemia, nutrients, mexico, infant, pregnancy

## Abstract

**Introduction:**

Maternal dietary consumption during pregnancy has been inconclusively associated with acute leukemia (AL) in infants, probably because epidemiological evidence has emerged mainly from the analysis of one-by-one nutrient, which is not a real-life scenario. Our objective was to evaluate the association between AL in Mexican children under 2 years of age and their mothers’ nutrients concomitant intake during pregnancy, as well as to explore whether there are differences between girls and boys.

**Methods:**

We conducted a study of 110 cases of AL and 252 hospital-based controls in the Mexico City Metropolitan area from 2010 to 2019. We obtained information on maternal intake of 32 nutrients by a food frequency questionnaire and used weighted quantile sum regression to identify nutrient concomitant intakes.

**Results:**

We found a concomitant intake of nutrients negatively associated with AL (OR 0.17; CI95% 0.03,0.88) only among girls; and we did not find a nutrient concomitant intake positively associated with AL.

**Discussion:**

This is the first study that suggests nutrients that have been individually associated with AL are not necessarily the same in the presence of other nutrients (concomitant intake); as well as that maternal diet might reduce AL risk only in girls.

## Introduction

Among pediatric cancers, acute leukemias (AL) are the most common with about one third of all cases and by its origin, frequency and behavior might be divided in lymphoid (Acute Lymphoid Leukemia: ALL) (85% of cases) and myeloid (Acute Myeloid Leukemia: AML) principally; from which little is known about the risk factors associated with the disease (1). Factors that have been positively related to this leukemias are ionizing radiation during pregnancy, high birth weight, sex, late viral infection, as well as possibly exposure to pesticides (2, 3). In contrast, breastfeeding and early infections have been negatively associated with this disease (4).

Acute leukemias are more frequent in boys compared to girls. Although the etiology of this difference remains unclear, it has been proposed that it might be partially explained by higher birthweight and number of childhood infections among boys, as well as by differential gene expression between sexes (5–7) which might result in a lower AL risk among girls. Despite the above, there are scarce reports that have considered potential sex differences when evaluating AL risk factors (8).

The peak incidence of this pathology occurs between 2 and 5 years of age, and it has been suggested that those cases diagnosed during the first months of life are biological and clinically different from those developed later. This early AL could be mostly related to prenatal exposures, including diet (3, 9). Maternal diet during pregnancy may influence the development of AL through its role in fetal development, including DNA synthesis and repair, the development of epigenetic processes, and the establishment of the infant’s immune system, which begins early in gestation (10).

Most of the existing epidemiological studies regarding AL have focused on the evaluation of folic acid intake, including the use of dietary supplements during pregnancy. In addition, iron supplementation has also been evaluated in various studies, with inconsistent results to date (2, 3, 11–13). There is increasing evidence of the importance of other nutrients in prenatal development, which may also be involved in promoting or preventing the development of childhood leukemia (1, 10, 12). However, the available information (2, 3, 11–13) is based on the evaluation of one-a-time nutrient which is a limited approach, since the concomitant intake of food nutrients might determine the overall AL risk. We have identified only one epidemiological study that found a maternal food concomitant consumption of folate, methionine, and vitamins B, that was negatively associated with AL (1). Thus, research is warranted about the association of childhood leukemia and maternal concomitant intake of nutrients, as well as to explore that relationship by sex.

We evaluated the associations between dietary maternal nutrient concomitant intakes during pregnancy and childhood AL in Mexican children under 2 years of age and explored differences between girls and boys.

## Materials and methods

A hospital-based case-control study was carried out in the Metropolitan area of Mexico City from 2010 to 2019. The study sample comprised children that were identified in 9 public hospitals of second and third level of care. This study was conducted according to the guidelines laid in the Declaration of Helsinki and all procedures were approved by the Mexican Institute of Social Security (IMSS, by its acronym in Spanish) Scientific Research Committee with number 2010-785-064 and registered at each participating hospital. Prior to the interview, we obtained written informed consent from the parents to participate (14).

The cases were younger than 24 months with a diagnosis of AL and no Down syndrome, which was confirmed with bone marrow smears and histochemical tests (myeloperoxidase, sudan black B reaction, esterases, periodic Schiff reaction (PAS) and acid phosphatase), as well as immunophenotype. We identified 237 eligible patients within the participating hospitals. Out of the 237 eligible children, 127 were excluded because the mother refused to participate (n=11) or the dietary information was incomplete (n=116). The participation rate of this group was 46.4%, which corresponds to 110 children, of which 80.00% were diagnosed with lymphoid leukemia, 16.36% with myeloid and 3.64% had no information. For each case, we selected a control of the same sex and similar age ( ± 12 months), who was identified in a second level hospital, from the same health institution where the cases were gathered (IMSS, Secretary of Health, Secretary of Health of Mexico City, State of Mexico Institute of Health, Institute of Security and Social Services for State Workers). In total, we identified 276 eligible controls with a participation rate of 91.3% (n=252), since 24 mothers did not agree to participate. Controls were recruited from surgery (circumcision, hernioplasty, orchiopexy, tonsillectomy, etc.) and non-surgery services (intoxication, first and second level burns, etc.).

Mothers of the participant children were interviewed directly in the hospitals by previously trained personnel, regarding their sociodemographic and reproductive characteristics, as well as their diet during pregnancy. In addition, parents also provided their information about tobacco and alcohol perinatal consumption.

### Maternal diet

Mothers were asked to report dietary intake during the pregnancy of their participating child, through a semi-quantitative food frequency questionnaire (FFQ), which included 109 foods and beverages, as well as 7 local dishes. The reproducibility of this instrument was evaluated in Mexican women, to whom it was applied twice at an interval of one year, while its validity was estimated using 24-hour recall questionnaires at 3-month intervals as a reference. The details of this validation have been published previously (15).

According to the methodology suggested by Willett et al. (16), the food questionnaire includes 10 response options for frequency consumption ranging from “never” to “6 or more times a day”, as well as predetermined portions for each food as follows: a glass (for milk and wine), a cup (for yogurt, some fruits and vegetables, tea, juices, alcoholic and non-alcoholic beverages), a spoon (for oils, sour cream, sauces and nuts), a slice (for cheeses, some fruits and meats), a plate (for legumes and local dishes) and a piece (for some fruits and breads).

We created a database with the nutritional and energy content of each food contained in the FFQ, which we obtained from the nutritional composition tables of the United States Department of Agriculture (17, 18), which includes a wide variety of foods that are consumed in Mexico. For local foods, such as tejocote, that were not found in those tables, we used the reference tables of the National Institute of Health Sciences and Nutrition “Salvador Zubirán” (19). In addition, two foods (soy juice and soy beer) were not found in any of the above tables, thus were not included in the subsequent calculation of total energy intake.

We calculated the daily consumption of kilocalories and nutrients of the mothers by summing those provided by each food. Because some fruits and vegetables are only consumed during certain seasons of the year, we adjusted their energy intake according to their availability on the market, for example, we only considered 50% of plum consumption, since it is only available during 6 months of the year (20).

We eliminated one participating case, because the estimated maternal total energy intake was less than 525 kcal, which correspond to minus 2 standard deviations of the daily intake observed in a study among pregnant Mexican women (21). Therefore, the final sample size was 109 cases and 252 controls.

### Statistical analysis

**Figure 1:**
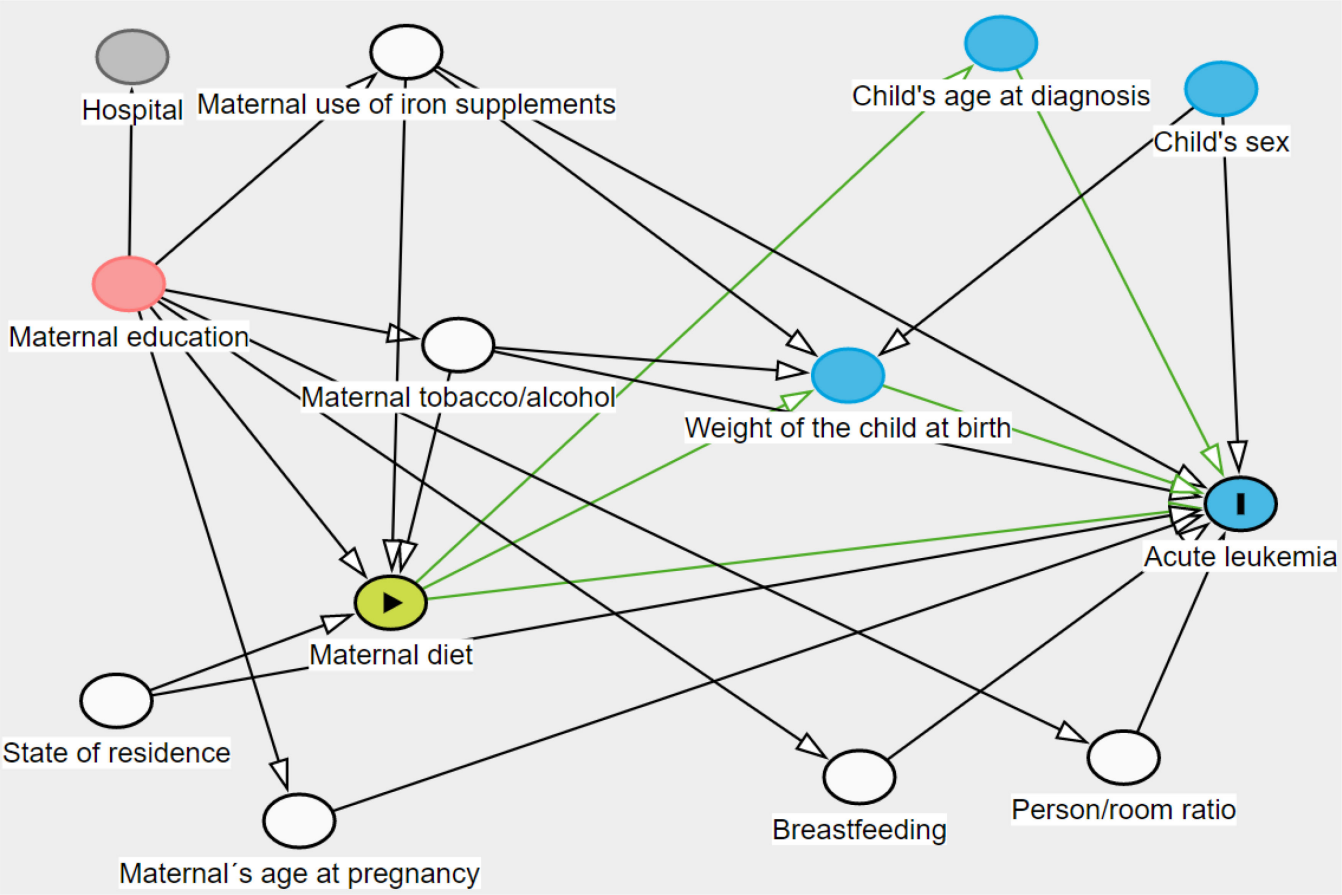
Directed acyclic graph of maternal diet intake and acute leukemia. White circles= adjusting variables; red circles= ancestor of exposure and outcome that does not need to be adjusted for; blue circle other than acute leukemia circle= ancestor of outcome. Green lines correspond to direct and indirect causal paths between maternal diet and Acute Leukemia, the latter are mediated by child’s age at diagnosis and weight of the child at birth.

We contrasted maternal education between included and non-included cases and controls. In addition, we compared selected characteristics, as well as energy and nutrient intakes between cases and controls using T-student, Mann Whitney U, Chi2, Fisher or ANOVA test. Each energy adjusted (22) nutrient intake was log-transformed and classified as quintiles based on controls distribution. We selected potential confounders through directed acyclic graphs (DAGs) (**Figure 1**). Through unconditional logistic regression models, we estimated the association between AL and each maternal nutrient intake, and we further stratified them by the child sex.

Common statistical techniques such as linear or logistic regressions are used when nutrients are analyzed with the one-by-one approach, however, since nutrients consumed through foods are highly correlated, other statistical techniques are required to allow them to be simultaneously evaluated despite their collinearity, as is the case of the weighted quantile sum regression (WQS). To assess the association of AL and the concomitant maternal nutrients intake by sex, as well as to identify the relative importance of each nutrient, we used WQS with binomial family specification, which identifies a unidirectional weighted index from quantiled nutrient variables and allowed us to identify the nutrients of concern in that index, while reducing the dimensionality and potential multi-collinearity observed among them. We constructed two indexes, one constrained to be positive and other constrained to be negative, with 50 bootstrap samples for each. Nutrients with mean weights, which indicate the relative strength of each component within the mixture, exceeding 3% (1/32 nutrients) were identified as nutrients of concern, indicating a larger contribution to the outcome than expected by chance. In addition, we applied repeated holdout validation, a method that combines cross-validation and bootstrap resampling. Specifically, we randomly split data into 40% training and 60% validation sets and repeated WQS regression 50 times to simulate a distribution of validated results from the underlying population. Finally, we reported mean weights per nutrient and characterized the uncertainty of these weights using a weight uncertainty plot (23, 24). In addition, we assessed in previous models, a multiplicative interaction term between the negative concomitant intake (continuous) and sex (categorical), as well as between the positive concomitant intake (continuous) and sex (categorical).

As a sensitivity analysis, we re-run the models, additionally adjusting by type of delivery, total number of children and birth order of the index child. In addition, we performed the models without the interaction term, excluding participants whose mothers reported, in the interview, to believe that diet is related to infant leukemia. To assess if controls diagnostics were associated with their maternal macronutrients and energy intake, we grouped them and compared them according to the type of clinical service: surgery (n=163) or non-surgery (n=88).

We evaluated uncentered variance inflation factors (VIFs) in our full adjusted logistic regression models, which resulted in a mean VIF less than 10, indicating no high correlation among the independent variables included. We also performed the goodness-of-fit test for those models and found that they fitted reasonably well (*p*-value<0.05).

We set an alpha of 0.05 as the level of significance and performed all analyses in R (4.1.1) and Stata 14 (StataCorp, College Station, TX, USA).

## Results

We found no differences regarding maternal education between included and non-including cases and controls (Data not shown). In our study population, cases were younger and heavier than controls and there were more females. In addition, mothers of the cases had lower iron and alcohol consumption during pregnancy. We found no differences regarding father’s characteristics of interest. Families of cases had a lower person/room ratio and they mainly lived in the State of Mexico (**Table 1**).

**Table 1:** General characteristics of the study sample.

Characteristics	(n)	Cases	(n)	Controls	*P*-value
Child					
Sex, girls %	(57)	52.29	(100)	39.68	0.026
Age, months [mean ± SD]	(109)	15.46 ± 7.30	(252)	18.48 ± 9.63	0.004
Birth weight, grams [mean ± SD]	(109)	3,144.78 ± 400.69	(252)	2,938.07 ± 610.89	0.001
Breastfeeding, yes %	(96)	88.07	(226)	89.68	0.651
Breastfeeding, months [p50(p10,p90)]	(109)	6.00 (0.00,15.00)	(252)	7.00 (0.00,18.00)	0.242
Mother					
Age at pregnancy, years [mean ± SD]	(109)	25.94 ± 6.41	(252)	25.58 ± 6.33	0.615
Education, years [p50 (p10,p90)]	(109)	9.00 (6.00,15.00)	(252)	10.00 (7.00,13.60)	0.833
Smoking before pregnancy, yes %	(31)	28.44	(77)	30.56	0.687
Smoking during pregnancy, yes %	(4)	3.67	(7)	2.78	0.740
Alcohol consumption during pregnancy, yes %	(4)	3.67	(31)	12.30	0.011
Iron supplement during pregnancy, yes %	(76)	69.72	(216)	85.71	0.000
Vitamins supplement during pregnancy, yes %	(98)	89.91	(238)	94.44	0.119
Drug use for genital infection, yes %	(15)	13.76	(34)	13.49	0.945
Father					
Age at pregnancy, years [mean ± SD]	(105)	28.79 ± 7.87	(250)	28.85 ± 7.39	0.944
Education, years [p50 (p10,p90)]	(104)	10.50 (6.00,15.00)	(245)	9.00 (6.00,14.00)	0.226
Smoking before pregnancy, yes %	(48)	47.06	(141)	58.02	0.062
Alcohol consumption before pregnancy, yes %	(88)	85.44	(222)	90.61	0.158
Family					
State of residence, %					
Mexico City	(42)	38.53	(132)	52.38	0.016
State of Mexico	(67)	61.47	(120)	47.62	
Health institution, %					
Ministry of health*	(62)	56.88	(164)	65.08	0.139
Mexican Institute of Social Security	(47)	43.12	(88)	34.92	
Person/room ratio, [p50(p10,p90)]	(109)	2.50 (1.50,5.00)	(252)	3.00 (1.67,6.00)	0.047

*Includes Secretary of Health, Secretary of Health of Mexico City, State of Mexico Institute of Health (ISEM, by its acronym in Spanish), Institute of Security and Social Services for State Workers (ISSSTE, by its acronym in Spanish). Shadowed numbers correspond to case-control differences within the variable with a p value <0.05.

We also found a high proportion of mothers that did not comply with the dietary reference intake values for folate, iron, calcium, choline and potassium; on average these proportions were 76%, 92%, 68%, 83% and 85%, respectively (Data not shown). All daily nutrient intakes did not differ between studied mothers (**Table 2**). The Spearman correlation coefficients between all nutrients included in this report range from 0.93 between alpha carotene and beta carotene, to -0.63 between magnesium and monosaturated fats (Data not shown).

**Table 2:** Energy and nutrient intake in the study sample.

Nutrients/day	Reference intake values/day ^a^	% of sample below		Energy and nutrient daily intakes		*P*-value
		Cases	Controls	Cases	Controls	
				P50 (P10,P90)		
Energy, kcal	NA			2271.09 (1402.20,3425.84)	2364.81 (1460.25,3650.83)	0.479
Protein, g	71 g ^b^	38.53	45.24	78.08 (47.54,128.44)	74.41 (47.67,114.79)	0.332
Carbohydrates, g	175	11.93	6.35	306.59 (166.58,499.26)	305.31 (190.38,489.72)	0.961
Fiber, g	28	31.19	33.33	34.48 (17.74,51.33)	34.32 (19.05,56.02)	0.539
Saturated fats, g	NA			24.85 (14.35,36.75)	22.66 (14.95,34.56)	0.050
Monosaturated fats, g	NA			32.72 (20.12,53.14)	31.91 (21.18,46.45)	0.652
Polyunsaturated fats, g	NA			26.33 (15.07,46.80)	25.29 (17.10,45.87)	0.897
Cholesterol, mg	NA			277.79 (134.97,497.26)	264.38 (127.72,444.88)	0.159
Retinol, mcg	NA			292.56 (155.53,807.02)	246.78 (133.98,880.49)	0.093
Alpha carotene, mcg	NA			1300.83 (137.63,2367.48)	1306.67 (278.26,2399.87)	0.260
Beta carotene, mcg	NA			3900.32 (1301.29,7308.64)	3965.01 (1593.87,7645.98)	0.249
Thiamin, B1, mg	1.4	29.36	25.40	1.68 (0.97,3.28)	1.73 (1.05,2.86)	0.499
Riboflavin, B2, mg	1.4	16.51	19.84	2.01 (1.21,3.80)	1.90 (1.08,3.17)	0.207
Niacin, B3, mg	18	46.79	45.63	18.85 (10.83,32.55)	18.71 (11.26,30.47)	0.586
Pyridoxine, B6, mg	1.9	39.45	39.68	2.09 (1.13,3.45)	2.13 (1.21,3.64)	0.499
Folate, mcg	600	78.90	73.02	411.26 (253.45,761.83)	445.81 (267.07,770.73)	0.153
Vitamin B12, mcg	2.6	10.09	17.86	4.67 (2.46,9.26)	4.55 (2.13,11.18)	0.824
Vitamin C, mg	85	14.68	13.10	136.96 (72.92,303.12)	164.70 (73.91,346.25)	0.110
Alpha tocopherol, mg	NA			12.14 (8.01,21.08)	13.02 (8.97,19.45)	0.090
Vitamin K, mcg	90	40.37	39.51	105.46 (48.87,239.62)	110.48 (50.28,289.56)	0.275
Choline, mg	450	82.57	84.13	334.93 (199.15,488.57)	324.35 (187.07,486.27)	0.372
Calcium, mg	1000	64.22	72.62	865.59 (476.66,1588.50)	823.54 (438.22,1346.87)	0.076
Phosphorus, mg	700	1.83	3.97	1725.75 (991.11,2637.98)	1528.02 (936.46,2537.78)	0.085
Magnesium, mg	350-360 *	43.12	48.02	386.05 (218.54,616.16)	367.31 (218.63,588.96)	0.646
Iron, mg	27	90.83	93.25	13.97 (8.17,26.55)	15.01 (8.84,25.10)	0.193
Zinc, mg	11	38.53	46.03	12.17 (6.93,18.48)	11.31 (6.74,20.13)	0.432
Copper, mg	1	12.84	15.48	1.52 (0.89,2.83)	1.56 (0.91,2.91)	0.737
Sodium, mg	1500	5.50	3.97	2866.48 (1676.82,4731.28)	2861.20 (1770.68,4579.40)	0.714
Potassium, mg	4700	85.32	85.32	3259.95(1951.05,5162.42)	3289.71 (1983.73,5104.27)	0.857
Caffeine, mg	NA			38.05 (2.99,166.39)	52.30 (9.23,86.17)	0.264
Theobromine, mg	NA			8.75 (1.34,20.42)	8.75 (1.34,20.42)	0.302
Methionine, g	NA			1.59 (0.95,2.51)	1.50 (0.92,2.32)	0.113
Betaine, mg	NA			29.81 (12.85,60.44)	32.39 (13.77,55.69)	0.147

^a^ Dietary Reference Intakes from the IOM, 2005; ^b^ based on g protein per kg of body weight for the reference body weight, *360 was selected as the comparison value. NA, not available.

After adjusting by state of residence (Mexico City & State of Mexico), person/room ratio, breastfeeding (months), maternal age at pregnancy (years), maternal tobacco (yes/no), and alcohol consumption (yes/no), as well as the intake of supplements of iron (yes/no) and vitamins (yes/no) during pregnancy, we observed that each quintile increase of maternal intake of saturated fat (OR=1.28; 95% CI 1.08,1.52), phosphorous (OR=1.23; 95% CI 1.04,1.46) and methionine (OR=1.23; 95% CI 1.03,1.46) was positively associated with AL. In contrast, the consumption of thiamine (OR=0.83; 95% CI 0.70,0.99), niacin (OR=0.77; 95% CI 0.65,0.92), pyridoxine (OR=0.77; 95% CI 0.64,0.93), folate (OR=0.80; 95% CI 0.67,0.94), vitamin C (OR= 0.84; 95% CI 0.71,0.99), alpha tocopherol (OR= 0.81; 95% CI 0.68,0.96) and iron (OR=0.74; 95% CI 0.62,0.89) was negatively related to this cancer. Interestingly, all these associations, except methionine, remained only among girls, for whom other negative associations emerged (copper and betaine). We also observed that among boys, riboflavin was the only nutrient (positively) related to AL (**Table 3**).

**Table 3:** Association between Acute Leukemia and maternal nutrient intakes during pregnancy by child sex.

Quintiles of daily nutrients intake	All	Boys	Girls
	OR (95% CI)*		
Protein, g	1.13 (0.96,1.34)	1.12 0.89,1.40)	1.07 (0.81,1.41)
Carbohydrates, g	0.86 (0.73,1.02)	0.98 (0.78,1.23)	0.76 (0.57,1.01)
Fiber, g	0.86 (0.72,1.02)	0.91 (0.72,1.15)	0.76 (0.56,1.03)
Saturated fat, g	**1.28 (1.08,1.52)**	1.16 (0.93,1.45)	**1.49 (1.09,2.04)**
Monosaturated fat, g	1.08 (0.91,1.27)	1.03 (0.81,1.30)	1.07 (0.82,1.40)
Polyunsaturated fat, g	0.92 (0.77,1.09)	0.86 (0.67,1.10)	1.05 (0.80,1.37)
Cholesterol, mg	1.19 (0.99,1.42)	1.27 (0.99,1.62)	1.05 (0.78,1.41)
Retinol, mcg	1.13 (0.95,1.35)	1.22 (0.96,1.56)	1.00 (0.75,1.34)
Alpha carotene, mcg	0.97 (0.81,1.15)	1.07 (0.84,1.36)	0.89 (0.67,1.18)
Beta carotene, mcg	0.89 (0.75,1.05)	1.02 (0.80,1.30)	0.78 (0.59,1.04)
Thiamine, B1, mg	**0.83 (0.70,0.99)**	1.06 (0.83,1.35)	**0.58 (0.43,0.79)**
Riboflavin, B2, mg	1.15 (0.97,1.36)	**1.33 (1.04,1.69)**	0.93 (0.71,1.23)
Niacin, B3, mg	**0.77 (0.65,0.92)**	0.95 (0.75,1.21)	**0.57 (0.41,0.78)**
Pyridoxine, B6, mg	**0.77 (0.64,0.93)**	0.87 (0.69,1.12)	**0.54 (0.38,0.76)**
Folate, mcg	**0.80 (0.67,0.94)**	1.00 (0.79,1.25)	**0.55 (0.40,0.76)**
Vitamin B12, mcg	0.99 (0.83,1.17)	1.10 (0.87,1.39)	0.76 (0.56,1.03)
Vitamin C, mg	**0.84 (0.71,0.99)**	1.02 (0.80,1.28)	**0.63 (0.47,0.86)**
Alpha tocopherol, mg	**0.81 (0.68,0.96)**	0.87 (0.70,1.10)	**0.68 (0.51,0.91)**
Vitamin K, mcg	0.91 (0.77,1.09)	0.96 (0.75,1.22)	0.79 (0.58,1.07)
Choline, mg	1.11 (0.93,1.32)	1.25 (0.98,1.59)	0.88 (0.66,1.17)
Calcium, mg	1.13 (0.95,1.33)	1.14 (0.91,1.42)	1.07 (0.81,1.42)
Phosphorus, mg	**1.23 (1.04,1.46)**	1.10 (0.88,1.38)	**1.36 (1.02,1.82)**
Magnesium, mg	1.03 (0.87,1.22)	1.00 (0.79,1.26)	1.00 (0.75,1.33)
Iron, mg	**0.74 (0.62,0.89)**	0.96 (0.76,1.22)	**0.47 (0.34,0.67)**
Zinc, mg	1.06 (0.90,1.25)	0.97 (0.77,1.22)	1.06 (0.81,1.40)
Copper, mg	0.86 (0.72,1.02)	1.01 (0.80,1.29)	**0.69 (0.52,0.92)**
Sodium, mg	0.95 (0.80,1.12)	1.12 (0.89,1.41)	0.75 (0.55,1.00)
Potassium, mg	0.89 (0.75,1.05)	0.93 (0.74,1.17)	0.77 (0.57,1.03)
Caffeine, mg	0.87 (0.73,1.03)	0.89 (0.70,1.12)	0.86 (0.65,1.14)
Theobromine, mg	1.02 (0.86,1.21)	1.00 (0.79,1.27)	1.16 (0.87,1.55)
Methionine, g	**1.23 (1.03,1.46)**	1.14 (0.91,1.43)	1.25 (0.93,1.69)
Betaine, mg	0.90 (0.76,1.06)	1.05 (0.83,1.33)	**0.73 (0.55,0.98)**

*Adjusted by state of residence, person/room ratio, breastfeeding, maternal age at pregnancy, as well as maternal tobacco and alcohol consumption and supplement use of iron and vitamins during pregnancy. Bold and shadowed numbers correspond to case-control OR differences > or <1.00 with P-value <0.05.

**Figure 2:**
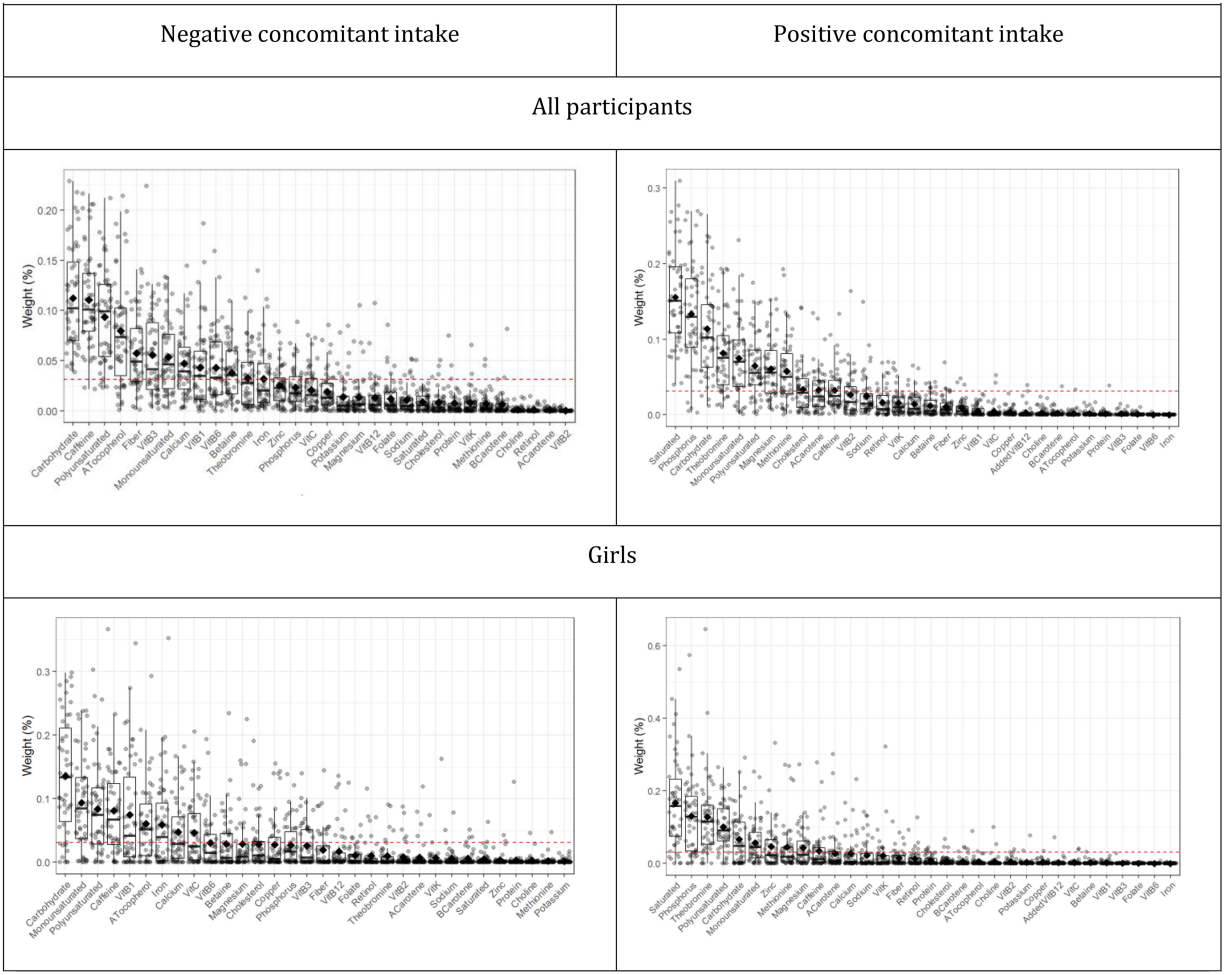
Identification and uncertainty of nutrients within the concomitant intakes associated with Acute Leukemia. Dotted red lines show the 0.03 threshold, identifying those nutrients of concern. Data points indicate weights for each of the 50 holdouts. Box plots show 25th, 50th, and 75th percentiles, and whiskers show 10th and 90th percentiles of weights for all holdouts. Diamonds show mean weights.

The WQS analysis, which included 32 nutrients of interest, showed 1 positive and 1 negative concomitant intake, in the whole sample and among girls. This analysis did not converge among boys, due to a lack of sufficient relevant association estimator (given by their magnitude and standard error) between each nutrient and AL. Both in the total sample and among girls, the negative concomitant intake was mainly characterized by carbohydrates, caffeine, thiamine, alpha tocopherol, calcium, iron, pyridoxine, betaine, mono and polyunsaturated fats, as well as by vitamin C among girls only. Likewise, the positive concomitant intake was characterized by phosphorus, theobromine, carbohydrates, methionine, magnesium, caffeine, saturated, mono and polyunsaturated fats, as well as cholesterol and alpha carotene in the whole sample only and zinc among girls only (**Figure 2**).

The estimated odds ratio between AL and the negative nutrient concomitant intake was 0.23 (CI 95% 0.15, 0.71) among all participants, which remains only among girls (OR 0.17; CI 95% 0.03, 0.88). Likewise, we observed an odds ratio of 1.55 (CI 95% 0.68,3.56) and 1.00 (CI 95% 0.49,2.02) between the positive concomitant intake and AL in the whole sample and among girls, respectively. In addition, we found that the multiplicative interaction term between sex and the negative concomitant intake had an odds ratio of 0.13 (CI 95% 0.02,074), while that for the positive concomitant intake was 1.19 (CI 95% 0.24,5.80) (**Table 4**).

**Table 4:** Association between Acute Leukemia and maternal nutrient concomitant intakes by child sex.

	(Ca, Co)	Nutrient concomitant intakes	
		Negative	Positive
		OR (95% CI)	
All	(109, 252)	**0.23 (0.15,0.71)**	1.55 (0.68,3.56)
Girls	(57, 100)	**0.17 (0.03,0.88)**	1.00 (0.49,2.02)
Boys	(52, 152)	–	–
Interaction	(109, 252)	**0.13 (0.02,0.74)**	1.19 (0.24,5.80)

In the body of the table, we show associations (odds ratios and 95% confidence intervals) between the respective concomitant intake (positive or negative) and childhood leukemia in the whole sample and by sex, that were adjusted by: residence, person/room ratio, breastfeeding, maternal age at pregnancy, as well as maternal tobacco and alcohol consumption and supplement use of iron and vitamins during pregnancy. In the case of boys, the above models did not converge due to lack of sufficient relevant odds ratios (given by their magnitude and standard error) between each nutrient and acute leukemia. On the bottom line of the table, the odds ratios and 95% confidence intervals of the interaction term are presented between: negative (continuous) concomitant intake and sex (categorical), as well as positive (continuous) concomitant intake and sex (categorical). Each energy adjusted nutrient intake was log-transformed and classified as quintiles. Bold and shadowed numbers correspond to case-control OR differences > or <1.00 with P-value <0.05.

The above results did not change when we additionally adjusted by type of delivery (vaginal or caesarean), total number of children (>2 vs. ≤2) and birth order of the index child (>2 vs. ≤2), neither when we excluded 9 participants whose mothers reported to believe that diet causes infant leukemia. Also, we did not observe differences in energy and macronutrient intakes between types of clinical services from controls: surgery versus non-surgery (Data not shown).

## Discussion

Our results suggest that individual and concomitant intake of some nutrients was negatively associated with AL in girls. In contrast, in boys we only identified an individual positive association between riboflavin consumption and AL. In addition, we did not observe a nutrient concomitant intake positively associated with AL, regardless of sex.

Among girls, we observed that AL was negatively associated with individual intake of thiamin, niacin, pyridoxine, folate, vitamin C, alpha tocopherol, iron, copper, and betaine; and positively related to saturated fats and phosphorus. In the same way, we identified a concomitant intake of nutrients negatively associated, which was characterized by carbohydrates, mono/polyunsaturated fats, caffeine, thiamine, alpha tocopherol, iron, calcium, vitamin C, pyridoxine, and betaine.

Several nutrients that were negatively associated with AL in this report, both individually and in concomitant intakes (thiamin, niacin, pyridoxine, betaine), are key elements that, together with folate, participate in one carbon metabolism resulting in nucleotides formation, as well as DNA methylation (1, 25). The relationship between the consumption of these nutrients and AL is complex, and probably depends on the dose and timing of their consumption. For example, it has been observed that the consumption of folate before the onset of cancer may reduce the risk of this disease. However, once the cancer is present, this nutrient might accelerate its evolution when consumed in high doses (26). Interestingly, in this report, folate was only individually associated with AL but did not have a relevant weight in the presence of other nutrients. From a statistical point of view, this result might indicate that the individual relationship of folate with this cancer does not reflect the potential correlations, as well as the synergistic or antagonistic interactions among nutrients. In this regard, in most (2, 3, 11–13, 27) of the previous reports that have evaluated the relationship between folate and AL, the consumption of other nutrients potentially related to this cancer has not been considered. However, in a study by Singer et al. (1), folate along with methionine and vitamins B2, B6 and B12 were found to be negatively associated with AL, using Principal Component Analysis. Although this statistical technique identifies concomitant intakes based on the correlation between nutrients, it does not consider their *a priori* association with the outcome (28), as the WQS method does. Therefore, further investigation is warranted to clarify the relationship between AL and folate, in the presence of other nutrients.

In addition, the antioxidant activity and/or the promotion of the immune response might explain the negative associations between AL with some B vitamins, as well as with vitamin C consumption (1, 29, 30). It has been suggested that the latter could also increase circulating folate levels, although the underlying biological mechanism remains poorly understood (30).

We observed that AL was negatively related to individual and concomitant intake consumption of alpha tocopherol, and iron. Previous epidemiological studies have not found a relationship between AL and iron, neither as a supplement (3) nor as dietary intake (12, 27). Iron participates in normal cell maintenance through the production of ATP, oxygen transport, and the synthesis of DNA; however, the excess of this nutrient might promote the development of leukemia due to its pro-oxidant activity (31). Nevertheless, in our study sample, less than 10% of the participants had an iron intake above its reference value, thus the probability of having many participants with excess dietary consumption of this nutrient is low. For its part, alpha tocopherol is an antioxidant that might stimulate the immune system (32, 33). However, we have not found evidence of its previous evaluation in epidemiological studies, thus, our results need to be confirmed.

Calcium and caffeine also characterized the concomitant intake of nutrients negatively associated with AL, although they were not individually related to this cancer, possibly due to their low consumption in the study sample. In this regard, about 68% of the participants had a calcium intake below their reference value, while the consumption of coffee was almost 3 cups a week (Data not shown). Calcium might reduce cell proliferation by increasing their programmed death (34); while caffeine has antioxidant capacity and ability to regulate DNA repair and the immune system (35). However, caffeine might induce DNA double-strand breaks and chromosomal translocations due to its capacity as a topoisomerase II inhibitor (36). Two previous epidemiological studies found no association between AL and calcium (12, 27). In addition, in a cohort study in which 141,216 participants with 96 children with AL were evaluated, no association was found between maternal coffee consumption and AL (37), although case-controls studies have reported positive associations (38, 39). Likewise, mono and polyunsaturated fats were important nutrients within the concomitant intake negatively associated with AL. Although some of these nutrients might have prooxidant activities, others have shown to promote antioxidant capacity, and cell membrane fluidity (40), as well as induce a decrease in leukemia cell lines viability (41). In two epidemiological studies found in this regard, no association was reported between AL and mono/polyunsaturated fats (12, 27), highlighting the need for further research.

In contrast, AL was positively associated with the consumption of saturated fat and methionine, respectively. These nutrients have not been linked to AL in previous epidemiological studies (1, 12, 27). It has been suggested that excess methionine could lead to DNA hypermethylation, inappropriate gene silencing, and abnormal histone methylation (42, 43). The recommended daily dose of methionine for pregnant women has been set in combination with cysteine, which is 25 mg/kg/day (44), so that a person of 70 Kg requires an intake of about 1.8 g of methionine/cysteine per day. Taking this value as a reference, around 24% of the participating mothers had a high intake of methionine (greater than 1.8 g of methionine per day). For its part, the consumption of dietary fats might reduce the synthesis of B vitamins and folate, since it induces a disturbance in the microbial balance (2, 41, 45). We also observed that the individual consumption of phosphorus was positively associated with AL, as it was found in a previous report, but without statistical significance (12). The positive relationship between phosphorus and AL could be explained since it might enhance gene expression, protein translation and cell proliferation rate (46, 47).

Additionally, we observed that, among male children, only maternal consumption of riboflavin was positively associated with AL. This relationship was also observed in an epidemiological report that included US boys and girls, although this did not reach statistical significance (12). It has been suggested that riboflavin might promote increased proliferation, invasion, and migration of cancer cells (48), however, we believe that this result might be a chance finding.

It is very important to highlight that our results show that the nutrients that are associated with AL in the one-by-one nutrient analysis do not necessarily remain when they are analyzed in the presence of other nutrients. For example, individual folate intake has been negatively associated with AL, both in previous (2, 3, 11–13) and in this study, but this association did not remain when we performed the nutrient concomitant intake analysis. From a biological point of view, this first finding suggests that one or several nutrients might interact antagonistically with folate in its association with AL, such as caffeine consumption. For example, it has been suggested that serum folate concentrations decrease with increased caffeine intake (49), which is compatible with the antagonistic interaction between these nutrients that we observed in our study sample (*p* for interaction <0.05) (Information not shown). On the other hand, statistically speaking, the high correlation between nutrients might underestimate its weight in the concomitant intake analysis (50). In this regard, we observed high correlations between folate and other nutrients such as vitamin B1 (Spearman coefficient=0.80) and iron (Spearman coefficient= 0.74). In contrast, some nutrients that individually did not show an association with AL, emerged associated in the concomitant intake analysis: carbohydrates, calcium, caffeine, monounsaturated and polyunsaturated fats. In this context, further research is needed to disentangle the underlying biological mechanisms between the concomitant intake of nutrients and AL.

Interestingly, our data suggest that maternal diet is related to AL mainly among girls, possibly because they have a more efficient one-carbon metabolism than boys. For example, it has been observed that the polymorphism of the methylenetetrahydrofolate reductase (MTHFR) C677T gene, might be about 2 times more frequent among boys. This polymorphism reduces the activity of the MTHFR enzyme by up to 70% (51, 52), which might result in a suboptimal use of the nutrients that participate in the metabolism of one carbon.

Another potential mechanism involves the immune system. It has been observed that girls have higher innate and adaptive immune responses than boys, probably because the X chromosome houses a large number of immune-related genes (5). Therefore, the potential to stimulate the immune system with some nutrients, like alpha tocopherol (32, 33), caffeine (35) vitamin B and C (1, 29, 30), might be greater among girls. In addition, studies in adults have suggested that women have a better ability to methylate DNA, possibly due to the stimulating effect of estrogen on the synthesis of choline, which participates in the remethylation of homocysteine to methionine. Although this explanation does not seem to be relevant in infants (53), as in the case of this report.

It has also been proposed that the higher birthweight of boys compared to girls of the same gestational age (100–200 grams), might partially explain the observed greater frequency of this cancer among boys, since high birthweight increases the number of mitotic events and thus the frequency of somatic mutations in larger babies. However, there might also exist direct effects for sex and AL, not mediated by birthweight such as differences in gene expression (5). According to our DAG, our results are adjusted by birthweight by including maternal tobacco/alcohol consumption and iron supplement use in our multivariable models.

Our findings should be interpreted taking into account some methodological limitations. Due to the small sample size, we cannot exclude the existence of associations between other nutrients, individually or concomitant intake, with AL or specific types of it. The participation rate of the cases was low (46%); however, maternal education among the included and non-included cases was similar. In addition, we cannot rule out that the mothers of the children with AL reported lower alcohol consumption during pregnancy, which would explain the higher alcohol consumption among controls. However, it is unlikely that they would have differentially reported each of the nutrients under study. Furthermore, when we excluded 9 mothers who associated their diet during pregnancy with the infant’s AL, we did not observe changes in our results (Data not shown). Therefore, we believe that the possibility that the results are biased by a differential error is unlikely, except for alcohol consumption. However, due to non-differential measurement error in dietary reporting, not only our individual nutrient-AL associations are underestimated, but also, it might be possible that the interaction between nutrients concomitant intake and sex might be spurious (54). We did not have the information of the MTHFR polymorphism to confirm that the relationship between MTHFR variants and AL is modified by sex.

In contrast, a strength point in this work is that controls had a high participation rate (>90%) and when grouped according to their surgical or non-surgical services, we observed no differences regarding energy and macronutrient intakes. We observed that the median energy consumption of the controls (2275 kcal) seems to be slightly higher than that reported in a sample of pregnant women from Mexico City (2166 kcal) (55). In addition, the evaluation of maternal nutrient concomitant intake in relation to AL in infants, might identify important nutrients related to AL that may not be observed in a one-by-one approach.

Our results suggest that maternal diet during pregnancy is related to AL development and these findings might provide for the first time an explanation related to diet during pregnancy with the lower AL incidence among girls. However, our findings must be interpreted with caution and need to be confirmed in prospective and larger studies in other populations.

## Data availability statement

The original contributions presented in the study are included in the article/supplementary material. Further inquiries can be directed to the corresponding authors.

## Ethics statement

The studies involving humans were approved by The Mexican Institute of Social Security (IMSS, by its acronym in Spanish) Scientific Research Committee with number 2010-785-064 and registered at each participating hospital. The studies were conducted in accordance with the local legislation and institutional requirements. Written informed consent for participation in this study was provided by the participants’ legal guardians/next of kin.

## Author contributions

AM-O performed the data analysis and wrote the original draft. MP-S wrote the protocol, and contributed to design conception, data acquisition, and interpretation of results. LE-H, ED-A, JT, KS-L, RP-A, MV-A, RE, MM-M, AG-A, LR-V, JD-H, JM-G, AC-E, ML-C, SM-S, JR-G, NH-P, JF-B, JP-G, MR-V, DT-V, JO-D, AM-R, LG-C, CA-H, JF, JN-E, MR, HR-V, DD-R and SJ-M contributed to the development of the protocol and data acquisition. JM-A designed the study, contributed to the interpretation of results, reviewed and edited the writing of the manuscript. LL-C conceptualized this report and supervised the data analysis and writing of the manuscript. All authors contributed to the article and approved the submitted version.
